# Effect of Prophylactic Antifungal Protocols on the Prognosis of Liver Transplantation: A Propensity Score Matching and Multistate Model Approach

**DOI:** 10.1155/2016/6212503

**Published:** 2016-09-26

**Authors:** Yi-Chan Chen, Ting-Shuo Huang, Yu-Chao Wang, Chih-Hsien Cheng, Chen-Fang Lee, Ting-Jun Wu, Hong-Shiue Chou, Kun-Ming Chan, Wei-Chen Lee, Ruey-Shyang Soong

**Affiliations:** ^1^Department of General Surgery, Keelung Chang Gung Memorial Hospital, Keelung 204, Taiwan; ^2^School of Medicine, Chang Gung University, Taoyuan 259, Taiwan; ^3^Department of Liver and Transplantation Surgery, Linkou Chang Gung Memorial Hospital, Taoyuan 259, Taiwan; ^4^Department of Chinese Medicine, College of Medicine, Chang Gung University, Taoyuan 259, Taiwan; ^5^Community Medicine Research Center, Keelung Chang Gung Memorial Hospital, Keelung 204, Taiwan

## Abstract

*Background*. Whether routine antifungal prophylaxis decreases posttransplantation fungal infections in patients receiving orthotopic liver transplantation (OLT) remains unclear. This study aimed to determine the effectiveness of antifungal prophylaxis for patients receiving OLT.* Patients and Methods*. This is a retrospective analysis of a database at Chang Gung Memorial Hospital. We have been administering routine antibiotic and prophylactic antifungal regimens to recipients with high model for end-stage liver disease scores (>20) since 2009. After propensity score matching, 402 patients were enrolled. We conducted a multistate model to analyze the cumulative hazards, probability of fungal infections, and risk factors.* Results*. The cumulative hazards and transition probability of “transplantation to fungal infection” were lower in the prophylaxis group. The incidence rate of fungal infection after OLT decreased from 18.9% to 11.4% (*p* = 0.052); overall mortality improved from 40.8% to 23.4% (*p* < 0.001). In the “transplantation to fungal infection” transition, prophylaxis was significantly associated with reduced hazards for fungal infection (hazard ratio: 0.57, 95% confidence interval: 0.34–0.96, *p* = 0.033). Massive ascites, cadaver transplantation, and older age were significantly associated with higher risks for mortality.* Conclusion*. Prophylactic antifungal regimens in high-risk recipients might decrease the incidence of posttransplant fungal infections.

## 1. Introduction

Orthotopic liver transplantation (OLT) is the treatment of choice for patients with hepatocellular carcinoma, end-stage liver disease, and acute liver failure [1]. Despite advances in surgical techniques, availability of immunosuppressants, and evidence-based guidelines for perioperative management to improve the overall survival of transplant recipients, rejections and infections still affect early posttransplantation mortality. Although advances in immunosuppressants has decreased the incidence of organ rejections, recipients are at greater risk of infections [2]. The use of immunosuppressants has been found to affect host immunity, causing recipients to become susceptible to viral and fungal infections, and subsequently death, after OLT [2, 3]. In addition, several lines of evidence reveal that intensive care unit conditions, surgical techniques, type of transplantation, type of anastomosis method, massive blood transfusion, and prophylactic antibiotics and immunosuppressants are associated with posttransplant fungal infections [4]. Despite the advances in surgical techniques leading to reductions in intraoperative blood transfusions and surgical time in recent years, the incidence of invasive fungal infection (IFI) still ranges from 5% to 20% [2, 5, 6].

IFI is the major cause of mortality in the early posttransplantation state. IFI-related mortality in organ transplantation has been found to cause up to 77% of deaths, one of the major causes of early posttransplantation mortality [6]. Among IFIs,* Candida* species are the most common pathogens, followed by* Aspergillus* species. Before 2009, antifungal prophylaxis was controversial owing to the lack of direct evidence that it improved survival. However, a prophylactic antifungal regimen for transplant recipients at high risk of fungal infection was suggested by the evidence-based guidelines of the Infectious Disease Society of America (IDSA) in 2009 [7]. Nevertheless, there is little direct evidence with regard to prognosis after such prophylactic strategies. Thus, we conducted a retrospective hospital-based cohort study to investigate whether routine antifungal prophylaxis regimens reduce the risk of fungal infections in patients receiving OLT. In addition, we conducted a multistate model to investigate transition-specific risk factors.

## 2. Patients and Methods

### 2.1. Study Cohort

Patients undergoing OLT between January 2005 and September 2014 at the Chang Gung Memorial Hospital, Linkou, were enrolled retrospectively and were followed up until December 2015. All patients receiving either deceased or living donor livers (LDLT) were enrolled, and routine screening of infections following OLT was conducted. Routine culture from ascites and catheter were conducted perioperatively and sputum culture was conducted routinely for patients under mechanical ventilation. Patients with fungal infection before transplantation were excluded to prevent overestimation of the incidence. Clinical data, including age, sex, type of hepatitis, status of liver cirrhosis, model for end-stage liver disease (MELD) score, indication for OLT, type of OLT, microbiological screening results, and status of ascites after OLT, were collected. Ethical approval was obtained from the Committee of Ethics in Biomedical Research of Chang Gung Memorial Hospital, and the study conformed to the ethical guidelines of the 1975 Declaration of Helsinki.

### 2.2. Prophylaxis Strategy

Our intervention group underwent routine antifungal prophylaxis. At our institution, since 2009, a prophylactic antifungal regimen was routinely provided to transplant recipients with a high (>20) MELD score before undergoing transplantation. Perioperative prophylaxis consisted of ceftriaxone (2000 mg/day) in two divided doses plus ampicillin sodium (1000 mg/q6 h) adjusted by renal function for patients with a lower MELD score (≤20) and vancomycin HCl (15 mg/kg/dose q12 h) adjusted by renal function plus Tienam (imipenem (500 mg)/cilastatin (500 mg); 500 mg/q6 h) adjusted by renal function for patients with a high MELD score (>20). We used echinocandins, either anidulafungin (100 mg/day) or micafungin (100 mg/day), for fungal prophylaxis to prevent drug interactions between the antifungal agents and the immunosuppressants (calcineurin inhibitors) [8].

### 2.3. Diagnosis of Fungal Infection

Our primary outcome was risk for fungal infection in patients with liver transplantation. The diagnosis of fungal infection was based on positive culture data after OLT, which revealed a specific fungus or positive findings of yeast in the blood, wound, urine, catheter, or sputum. A positive fungal culture from urine combined with clinical manifestations was identified as a fungal infection instead of colonization. Positive cultures from blood, urine, and sputum depended on the clinical manifestation to define it as an infection.

### 2.4. Statistical Analysis

Continuous variables were summarized as median with interquartile range, while categorical variables were presented as frequency and percentage. In univariate analysis, baseline characteristics were compared between the intervention group and nonintervention group using the chi-squared test, Fisher's exact test, or Wilcoxon's rank-sum test, as appropriate. To reduce selection and confounding biases, we conducted propensity score matching using the nearest neighbor matching method with a 1 : 1 ratio for the intervention and nonintervention groups [9]. Furthermore, we used a multistate model to model the “transplant to fungal infection transition,” “transplant to death transition,” and “fungal infection to death transition,” occurring as a result of various reasons [10, 11]. First, death is a competing event with fungal infection occurrence. Second, we could simultaneously model all 3 transitions and estimate the cause-specific cumulative hazards, as well as cause-specific transition probability. In addition, we conducted cause-specific Cox models to investigate predictors of the 3 transitions. We performed model selection by Akaike information criterion (AIC) in a stepwise algorithm and substantive knowledge to find the parsimonious models [12]. In addition, we investigated the proportional hazards assumption using the modified Schoenfeld residuals test [13]. All reported confidence intervals (CIs) and tests were two-sided, with a 5% significance level. All analyses were performed using R software version 3.3.1 (R Foundation for Statistical Computing, Vienna, Austria) with contributed packages “MatchIt” [9], “MASS” [14], “mstate” [10, 11], and “survival” [13].

## 3. Results

A total of 561 patients were enrolled, of which 360 (64.2%) received the routine prophylactic antifungal regimen and 201 (35.8%) did not. After propensity score matching, a total of 402 patients were included for further analysis and the variables were comparable between the two groups. The demographic data before and after matching are presented in Table 1. After matching, the rate of fungal infection was 18.9% before routine prophylaxis and 11.4% after prophylactic treatment (*p* = 0.052), and the overall mortality rate of the recipients was 40.8% before the use of routine prophylaxis and 23.4% after (*p* < 0.001). Hepatitis B virus infection was dominant in the OLT recipients, followed by hepatitis C virus infection. The median time of fungal infection in the prophylaxis group was 27 days (interquartile range (IQR) 10.5–77.7 days), whereas it was 21 days (IQR 10–48.5 days) in the nonprophylaxis group. The transition matrix of the 3 states is summarized in the Supplemental Table 1 (see Supplementary Material available online at http://dx.doi.org/10.1155/2016/6212503). 61 of 402 (15%) patients developed “transplantation to fungal infection” transition. Among 61 patients with fungal infection, 36 (59%) patients died.

The species causing fungal infection are shown in Table 2. The most common fungal infection was by* Candida albicans*: 33.7% of infections before routine prophylaxis and 35.9% after.* Aspergillus* infection disappeared after initiating the routine prophylactic antifungal regimen. The incidence of* Candida glabrata* and* Candida tropicalis* increased after frequent echinocandin usage. Yeast was found in 32.3% and 28.2% of the cultures before and after prophylaxis, respectively. Eleven patients in the infected group developed 2 kinds of fungal infections and 1 developed 3 kinds of fungal infections.

**Figure 1:**
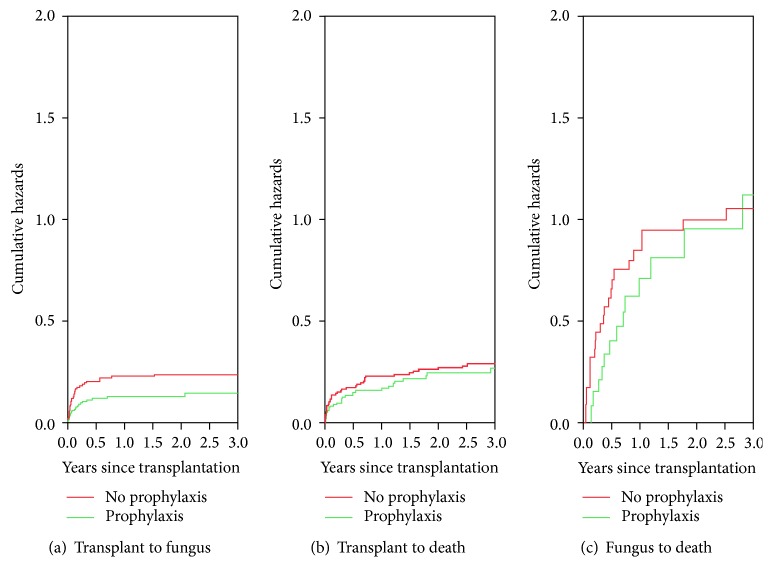
Nonparametric estimates of cumulative hazards of the multistate model stratified by transitions. (a) Cumulative hazards for the transition from liver transplantation to fungal infection demonstrate the fungal infection which occurred within the early period of transplantation and reached a plateau after 3 months. The routine prophylaxis group has lower cumulative hazards of “transplantation to fungal infection” transition. (b) Cumulative hazards for the transition from liver transplantation to death between the two groups were similar. (c) Cumulative hazards for the transition from fungal infection to death between the two groups were similar.

A multistate model was used to evaluate the cumulative hazards and transition probability after OLT for “transplantation to fungal infection,” “transplantation to death,” and “fungal infection to death” transitions.  Figure 1 shows that the cumulative hazards of a “transplantation to fungal infection” transition were lower in the routine prophylaxis group compared to the nonprophylaxis group. Cumulative hazards for “transplantation to death” and “fungal infection to death” transitions were similar in the 2 groups. We estimated 1-year, 2-year, and 3-year transition probabilities among the four states including “transplantation,” “fungal infection,” “death with fungal infection,” and “death without fungal infection” (Table 3). The routine prophylaxis group had a lower probability of “fungal infection” and “death with fungal infection.” Notably, the sum of “fungal infection” and “death with fungal infection” probability did not obviously increase over time, indicating most fungal infections occurred in the early posttransplantation period.

We also investigated predictors in 3 transition-specific multivariable Cox models (Table 4). In the transition from “transplantation to fungal infection,” the routine prophylaxis group was significantly associated with reduced hazards for fungal infection compared with the nonprophylaxis group (hazard ratio (HR): 0.57, 95% confidence interval (CI): 0.34–0.96, *p* = 0.033). In the transition from “transplantation to death,” patients with massive ascites were associated with a higher risk for mortality compared to patients with mild/moderate ascites (HR: 1.55, 95% CI: 1.02–2.36, *p* = 0.042). By checking the proportional hazards assumptions, LDLT was statistically significant associated with time-varying effects. Thus, we used the time point of 1.5 years after liver transplantation to model the LDLT effects (this time point was indicated by the residual plots). LDLT was associated with a lower risk for short-term mortality (with 1.5 years) compared to patients receiving cadaver transplantation (HR: 0.41, CI: 0.26–0.66, *p* ≤ 0.001). However, 1.5 years after liver transplantation, LDLT was not significantly associated with a lower risk for mortality. In the transition from “fungal infection to death,” older age and massive ascites were significantly associated with a higher risk of mortality.

## 4. Discussion

Our current study demonstrated that routine prophylactic antifungal regimens are associated with a lower risk for “transplantation to fungal infection” in patients receiving OLT. Patients with massive ascites had a higher risk for “transplantation to death” and “fungal infection to death” transitions. Patients receiving LDLT had a lower risk for the “transplantation to death” transition within 1.5 years after OLT.

Because of the evolution of surgical techniques and improvements in post-OLT management, the 5-year survival rate after OLT has reached 72–77% in recent times [15]. However, IFI is still one of the major causes of early mortality in liver transplant recipients. The incidence of IFI ranges from 5% to 20% [2, 5, 6]. According to the literature,* Candida* and* Aspergillus* are the most common causal agents and are associated with high mortality in organ transplantation, accounting for 30–60% of the infections [5, 16].

The reported risk factors for IFI include retransplantation, dialysis, prolonged operation time, and prolonged broad-spectrum antibiotics use [2, 17]. Fungal infections most frequently occur in the first month after OLT, and antifungal prophylaxis could significantly reduce fungal infections in patients receiving OLT. In a recent meta-analysis, antifungal prophylaxis has been shown to reduce fungal infection-related mortality [18]. Among antifungal drugs, echinocandins, such as caspofungin, micafungin, and anidulafungin, have excellent* in vitro* activity against* Candida* species, with few side effects and minimal drug-drug interactions; in particular, they do not influence the clearance of calcineurin inhibitors, which are commonly used immunosuppressants. Moreover, dosage adjustment is not required in patients with impaired renal function or those under dialysis [19].

Although advances in perioperative management and surgical techniques enable improved survival of OLT recipients, an infection after OLT is a major cause of mortality. A prophylactic protocol was established at our institution since 2009, which includes empiric antibiotics based on the MELD score and a regimen of prophylactic antifungal treatment in high-risk patients. IDSA guidelines have suggested prophylactic antifungal treatment in patients with renal dysfunction, retransplantation, or reoperation; however, more recent guidelines suggest prophylaxis in high-risk patients with high MELD scores, choledochojejunostomy, bile leaks, and LDLT [17, 20, 21]. Our current study showed that routine antifungal regimens reduce the risk for a “transplantation to fungal infection” transition. In addition, our results suggest fungal infections mostly occurred within the initial 3 months after OLT, which is consistent with other reports.

Most infections, either bacterial or fungal, occur in the first month after OLT, causing early mortality after transplantation in the first year [22]. In the current study, 1-year, 2-year, and 3-year probability of fungal infection and death with fungal infection were reduced in the prophylaxis group. These results indicate prophylactic protocols might reduce the incidence of fungal infection and death with fungal infection. The IDSA guideline in 2009 recommended routine antifungal prophylaxis for OLT recipients [23]. Recently, Saliba et al. reported that a MELD score of >30 might be the most important risk factor for IFI [17]. Patients with a MELD score > 20 have a higher possibility of pretransplantation renal dysfunction and liver dysfunction; therefore, we use this threshold for prophylaxis.

Patients undergoing OLT can have several time-dependent outcomes during follow-up [24]. A multistate model has been applied to analyze competing risks in patients with liver cirrhosis [25]. By evaluating the transition-specific risk factors from the multistate model, we found that a routine prophylactic antifungal regimen in high-risk recipients prevents further fungal infection. In patients with fungal infections,* C. albicans* was the most common species before and after prophylaxis. The incidence of other species was reduced after prophylaxis. Such findings are consistent with the literature [26]. Intrinsic resistance or resistance induced by the prophylactic agent might account for these findings. In addition, acquired resistance to echinocandins has been reported even in clinically relevant* Candida* spp. [27]. Acquired resistance species are associated with high mortality after fungal infections. Changing antifungal treatment from echinocandins to azoles or to liposomal amphotericin B should be considered if a positive fungal culture persists even after the antifungal regimen treatment. However, more data need to be collected to confirm this approach. In our institution, the initial positive culture of fungal infection revealed only yeast or molds, and further differentiation of the species required special cultures. If the patients' general condition improved after treatment or the condition became worse, we may not perform specific cultures for further differentiation and that is why almost one-third of the cultures revealed yeast only.

LDLT is associated with a lower short-term risk of “transplant to death” transition. The time-dependent effects might be associated with high-risk patients leaving the risk-set in the early period. LDLT might be associated with a shorter waiting time for the organ and prevention of the deterioration of liver function in the recipients. In addition, more reserve liver function and improved surgical techniques may improve long-term survival. Additionally, massive ascites indicates decompensated liver function and leads to about 50% mortality 2 years after patients present with uncontrolled ascites [28].

Our study has several advantages. First, we used propensity score matching to reduce selection and confounding biases in this observational study. Second, we used time to event outcomes in the current study. Such an approach allowed us to investigate time-varying treatment effects and adjust for competing risks (mortality is a competing risk for fungal infections, because it prevents the occurrence of fungal infection). If we did not adjust for competing risks, we would overestimate the cumulative incidence of fungal infection. Third, we used a multistate model to investigate the transition-specific risk factors.

Nevertheless, our present study has several limitations. First, unmeasured confounders could not be matched. For example, we could not match important biological data and donor-related factors. Second, time-lag bias can compromise the results in such a long-term observational study. For example, the reduced risk of fungal infection might be associated with more experience in surgical technique, improved intensive unit care, and better surgical facilities. Third, the small sample size in the current study limits us from thoroughly investigating predicting factors. Finally, this retrospective analysis of observational hospital-based cohort data might have information and performance biases.

## 5. Conclusion

We conclude that administering routine empiric antibiotic treatment and a prophylactic antifungal regimen to high-risk patients might reduce the incidence of fungal infection in the early stage after liver transplantation and prevent fungal infection-related mortality, which might lead to better long-term survival.* Candida* species remain the major cause of fungal infection despite prophylaxis. Further clinical trials are warranted to confirm our results.

## Supplementary Material

States of the patients receving liver transplantation include transition of transplantation to fungal infection, transplantation to death and fungal infection to death. The incidence of events happned are summaried in this table.

## Figures and Tables

**Table 1:** Demographic data of the liver transplantation recipients (before and after matching).

	Before matching			After matching		
	No prophylaxis	Prophylaxis	*p* value	No prophylaxis	Prophylaxis	*p* value
*N*	201	360		201	201	

Age (median (IQR))	53.00 (47.00, 57.00)	55.00 (48.00, 60.00)	0.021	53.00 (47.00, 57.00)	54.00 (48.00, 59.00)	0.228

Age (%)						
≤55	71 (35.3)	123 (34.2)	0.048	71 (35.3)	69 (34.3)	0.953
>55–60	101 (50.2)	155 (43.1)		101 (50.2)	101 (50.2)	
>60	29 (14.4)	82 (22.8)		29 (14.4)	31 (15.4)	

Sex (%)						
Male	153 (76.1)	264 (73.3)	0.533	153 (76.1)	160 (79.6)	0.471
Female	48 (23.9)	96 (26.7)		48 (23.9)	41 (20.4)	

HCC (%)						
No	112 (55.7)	195 (54.2)	0.79	112 (55.7)	108 (53.7)	0.764
Yes	89 (44.3)	165 (45.8)		89 (44.3)	93 (46.3)	

Viral hepatitis (%)						
None	26 (12.9)	78 (21.7)	0.017	26 (12.9)	29 (14.4)	0.905
HBV	126 (62.7)	183 (50.8)		126 (62.7)	127 (63.2)	
HCV	38 (18.9)	84 (23.3)		38 (18.9)	33 (16.4)	
HBV + HCV	11 (5.5)	15 (4.2)		11 (5.5)	12 (6.0)	

Ascites (%)						
Mild/moderate (≦2000 mL)	139 (69.2)	256 (71.2)	0.484	139 (69.2)	138 (68.7)	1
Massive (>2000 mL)	62 (30.8)	104 (28.9)		62 (30.8)	63 (31.3)	

Living donor (%)						
No	64 (31.8)	72 (20.0)	0.002	64 (31.8)	58 (28.9)	0.588
Yes	137 (68.2)	288 (80.0)		137 (68.2)	143 (71.1)	

MELD score						
≦20	130 (64.7)	258 (71.7)	0.104	130 (64.7)	133 (66.2)	0.834
>20	71 (35.3)	102 (28.3)		71 (35.3)	68 (33.8)	

Fungal infection (%)						
No	163 (81.1)	315 (87.5)	0.054	163 (81.1)	178 (88.6)	0.052
Yes	38 (18.9)	45 (12.5)		38 (18.9)	23 (11.4)	

Mortality (%)						
No	119 (59.2)	272 (75.6)	<0.001	119 (59.2)	154 (76.6)	<0.001
Yes	82 (40.8)	88 (24.4)		82 (40.8)	47 (23.4)	

Propensity score (median (IQR))	NA	NA		0.40 (0.32, 0.46)	0.40 (0.32, 0.44)	0.690

IQR: interquartile range, HCC: hepatocellular carcinoma, and MELD: model for end-stage liver disease. Ascites was measured during the operation.

**Table 2:** Species of fungus before and after the prophylactic anti-fungal protocol.

No prophylactic period			Prophylactic period		
Species	Number	%	Species	Number	%
*Candida albicans*	25	33.7	*Candida albicans*	28	35.9
*Candida glabrata*	7	9.9	*Candida tropicalis*	12	15.3
*Candida tropicalis*	5	6.7	*Candida glabrata*	5	6.4
*Candida parapsilosis*	4	5.4	*Candida parapsilosis*	3	3.8
*Candida krusei*	2	2.7	*Candida krusei*	1	1.3
*Aspergillus*	3	4.0	*Candida guilliermondii*	1	1.3
Mold	2	2.7	*Candida sp.*	1	1.3
*Penicillium sp.*	1	1.3	*Mucor sp.*	1	1.3
Trichosporon sp.	1	1.3	Mold	4	5.0
Yeast	23	32.3	Yeast	22	28.2

Note. Eleven patients developed 2 kinds of fungal infection and 1 developed 3 kinds of fungal infection. Mold and yeast species are not routinely identified without physician's requests.

**Table 3:** The 1-year, 2-year, and 3-year transition probability among four states in the multistate model.

	State occupied probability (95% CI)		
	1-year	2-year	3-year
*Prophylaxis*			
Transplant	74.2 (67.9–80.5)	68.1 (60.8–75.3)	65.4 (57.6–73.2)
Fungus infection	6.3 (2.3–9.7)	4.8 (1.8–7.9)	5.0 (1.5–8.4)
Death with fungus infection	5.5 (2.3–8.6)	6.9 (3.3–10.5)	7.9 (4.0–11.8)
Death w/o fungus infection	14.0 (9.0–19)	20.2 (13.8–26.6)	21.7 (14.8–28.6)

*No prophylaxis*			
Transplant	62.6 (55.9–69.4)	59.5 (52.7–66.3)	58.5 (51.6–65.3)
Fungus infection	10.3 (6.1–14.4)	9.2 (5.3–13.2)	8.7 (4.9–12.6)
Death with fungus infection	8.7 (4.9–2.5)	10.2 (6.1–14.3)	10.7 (6.5–14.9)
Death w/o fungus infection	18.4 (13.0–23.8)	21.0 (5.4–26.7)	22.1 (16.3–27.8)

**Table 4:** Results of multivariable transition-specific Cox models.

Variable	Category	HR (95% CI)	*p* value
*Transition: transplant to fungal infection*			
Treatment	No prophylaxis	1	
	Prophylaxis	0.57 (0.34–0.96)	0.033^*∗*^
Ascites	Mild/moderate	1	
	Massive	1.65 (0.98–2.76)	0.058
Propensity score		0.15 (0.01–1.76)	0.132

*Transition: transplant to death*			
Ascites	Mild/moderate	1	
	Massive	1.55 (1.02–2.36)	0.042^*∗*^
Living donor (within 1.5 years)	No	1	
	Yes	0.41 (0.26–0.66)	<0.001^*∗*^
Living donor (after 1.5 years)	No	1	
	Yes	1.08 (0.45–2.62)	0.861

*Transition: fungal infection to death*			
Age (years)	≤50	1	
	>50, ≤60	2.55 (1.10–5.93)	0.029
	>60	1.80 (0.67–4.83)	0.240
Ascites	Mild/moderate	1	
	Massive	2.19 (1.06–4.52)	0.035
Living donor	No	1	
	Yes	0.57 (0.28–1.14)	0.113

HR: hazard ratio and CI: confidence interval. ^*∗*^ *p* < 0.05.
